# Maternal weight in the postpartum: results from the Delta healthy sprouts trial

**DOI:** 10.1186/s40748-017-0058-9

**Published:** 2017-12-04

**Authors:** Lisa M. Tussing-Humphreys, Jessica L. Thomson, Nefertiti OjiNjideka Hemphill, Melissa H. Goodman, Alicia S. Landry

**Affiliations:** 1 Department of Medicine and Cancer Center, University of Illinois at Chicago, 416 West Side Research Office Building, 1747 West Roosevelt Road, Chicago, IL 60608 USA; 2 United States Department of Agriculture, Agricultural Research Service, Delta Human Nutrition Research Program, 141 Experiment Station Road, Stoneville, MS 38776 USA; 3 Department of Kinesiology and Nutrition, 484 West Side Research Office Building, 1747 West Roosevelt Road, Chicago, IL 60608 USA; 4 Department of Family and Consumer Sciences, University of Central Arkansas, 201 Donaghey Avenue, McAlister 113, Conway, AR 72035 USA

**Keywords:** Postpartum, Body weight, African American women

## Abstract

**Background:**

Excessive postnatal weight retention may pose a threat to a woman’s health and future pregnancies. Women in the Lower Mississippi Delta (LMD) region of Mississippi suffer from among the highest rates of obesity in the U.S. and are more likely to gain an excessive amount of weight during pregnancy. The aim of this study was to determine if LMD women who received a lifestyle enhanced maternal, infant, and early childhood home visiting (MIECHV) curriculum had more favorable weight outcomes through 12-months postpartum compared to women who received a standard MIECHV curriculum.

**Methods:**

Delta Healthy Sprouts was a two-arm, randomized, controlled, comparative impact trial. Pregnant women at least 18 years of age, less than 19 weeks pregnant with a singleton pregnancy, and residing in the LMD region were recruited. On a monthly basis in the participant’s home, the control arm (PAT) received the Parents as Teachers curriculum while the experimental arm (PATE) received a lifestyle enhanced Parents as Teachers curriculum. Pre-pregnancy body weight via self-report and maternal body weight at baseline (gestational month 4) and at every subsequent monthly visit through 12 months postpartum was measured. Linear mixed models were used to test for significant treatment, time, and treatment by time effects on postnatal weight outcomes.

**Results:**

Mean postnatal weight losses were 0.8 and 1.1 kg at postnatal month (PM) 6 and PM 12, respectively, for PAT participants. Mean postnatal weight losses for PATE participants were 1.5 and 1.2 kg at PM 6 and PM 12, respectively. Mean weight retention, based on pre-pregnancy weight, were 5.2, 4.0, and 3.6 kg at PM 1, PM 6, and PM 12, respectively, for PAT participants. Mean weight retention for PATE participants were 6.3, 4.5, and 4.0 kg at PM 1, PM 6, and PM 12, respectively. Significant effects were not found for treatment, time, or treatment by time.

**Conclusions:**

An enhanced MIECHV curriculum was not associated with more favorable postpartum weight outcomes when compared to a standard MIECHV curriculum in a cohort of LMD women during the 12 months following the birth of their infant. Trial registration: clinicaltrials.gov, NCT01746394. Registered 5 December 2012.

## Background

Pregnancy is a time when a woman intentionally gains weight to support her developing fetus and the pregnancy-related adaptations occurring including growth of the placenta. However, according to the Centers for Disease Control and Prevention, 48% of women in the United States (U.S.) gain an excessive amount of weight with a singleton pregnancy [1]. Specific weight gain recommendations for singleton pregnancies were put forth by the Institute of Medicine (IOM) in 2009 [2], and are based on a woman’s pre-pregnancy body mass index (BMI). The IOM established guidelines given mounting evidence that excessive weight gain in pregnancy can lead to significant transient and long-term health effects for both mother and baby. Specific to the mother’s postpartum health, excessive gestational weight gain is a risk factor for excessive postnatal weight retention (> 10 lb) [3], obesity [4], and obesity-related chronic diseases including type 2 diabetes [5]. Excessive postnatal weight retention may pose a threat to reproductive health and future pregnancies by increasing the risk for infertility [6], gestational diabetes [7] and pre-eclampsia [8]. Thus, perinatal lifestyle interventions promoting optimal gestational weight gain and postnatal weight management may have a significant effect on maternal and infant health.

Women of reproductive age in the Lower Mississippi Delta (LMD) region of Mississippi suffer from among the highest rates of obesity in the nation [9]. Furthermore, women residing in this area of the U.S. are likely to gain an excessive amount of weight during pregnancy [10] and have unintended, closely spaced pregnancies [11], both of which are associated with increased risk of obesity [12]. Hence, there is a dire need for perinatal weight management interventions in the LMD region.

Several lifestyle interventions focused on optimizing gestational weight gain have reported promising results [3, 13, 14], although only a few studies have spanned the perinatal period by actively intervening on both gestational weight gain and postnatal weight management [15–19]. Delta Healthy Sprouts was designed to test the impact of a maternal, infant and early childhood (MIECHV) home-visiting curriculum enhanced with a lifestyle recommendations (maternal and infant-related diet and physical activity and maternal weight management) compared to a standard MIECHV curriculum on maternal gestational weight gain and postnatal weight management amongst other infant and maternal health and behavioral outcomes in pregnant women residing in the LMD region [20]. We found that the lifestyle-enhanced MIECHV curriculum was not associated with more favorable gestational weight gain outcomes compared to the standard MIECHV curriculum [21]. Here, we present the 12-month postnatal weight management data for women enrolled in Delta Healthy Sprouts. The goal is to determine if the women who received the lifestyle enhanced MIECHV curriculum had more favorable weight outcomes through 12 months postpartum compared to the women who received the standard MIECHV curriculum.

## Methods

### Design and recruitment

This was a longitudinal analysis of Delta Healthy Sprouts maternal postnatal weight outcomes through 1 year postpartum. A full description the Delta Healthy Sprouts Trial has been reported previously [20]. Briefly, 82 pregnant women residing in the LMD region were enrolled in their second trimester of pregnancy. Inclusion criteria comprised female gender, 18 years or older, less than 19 gestational weeks with first, second, or third child, singleton pregnancy, and a resident of Washington, Bolivar, or Humphreys County in Mississippi. Women were recruited on a rolling basis between March 2013 and December 2014. Recruitment included active recruitment at local health clinics serving pregnancy women, publicizing the study in local print media and at local health fairs, referrals from the local health department and Women, Infants, and Children (WIC) staff, and by word of mouth.

**Fig. 1:**
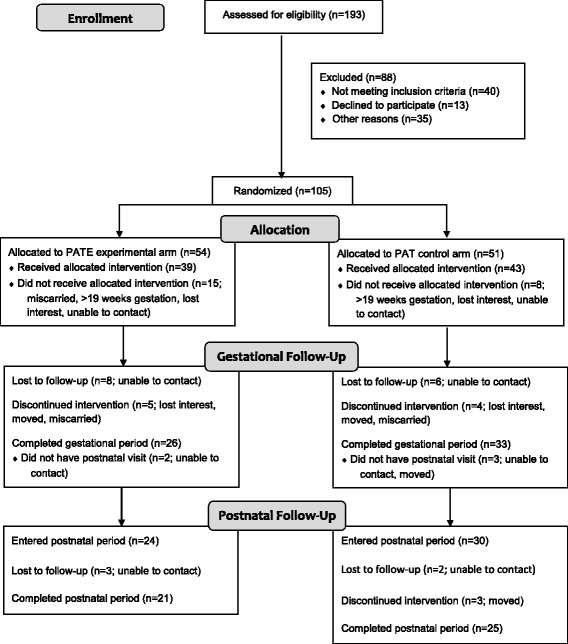
CONSORT Diagram for the Delta Healthy Sprouts Trial

The original recruitment goal was 75 women per treatment arm. The sample size of 150 women was based on the following assumptions: 20% attrition rate, 37% of control participants with gestational weight gain within the IOM recommendations, and a 22% difference between treatment arms for gestational weight gain within recommendations. Additionally, assuming an average 12-month postnatal weight loss of 1.5 kg in the PAT arm (SD = 4.7 and 5.4 kg in control and intervention arms, respectively) [22], a postnatal sample size of 120 participants would allow for detection of a 3.8 kg difference in 12-month postnatal weight loss between the two arms. An additional power and sample size calculation for the postnatal primary outcome – child obesity at 1 year of age – also was performed [20]. Recruitment was stopped prior to reaching the goal due to unanticipated difficulties with recruiting pregnant women meeting the study inclusion criteria and lack of resources to further support recruitment activities. All data collection activities concluded in May 2016. Figure 1 illustrates the CONSORT diagram for the study.

Delta Healthy Sprouts was designed to evaluate the impact of the MIECHV Parents as Teachers ® (PAT) curriculum compared with a lifestyle-enhanced PAT curriculum (PATE) on maternal gestational weight gain, postpartum weight management and childhood obesity prevention among other maternal and infant health and behavioral outcomes. PAT is a nationally recognized evidence-based MIECHV program that strives to increase parental knowledge of healthy child development, instill good parenting skills, provide early detection of physical and neurocognitive developmental delays, prevent child abuse, and increase school readiness [23]. Participants in the Delta Healthy Sprouts Trial were randomly assigned to one of the two treatment arms [PAT control (*N* = 43) or PATE experimental (*N* = 39)] at approximately 4 months gestation and followed through 12 months postpartum.

The Delta Healthy Sprouts Trial is registered at clinicaltrials.gov (NCT01746394) and was approved by the Institutional Review Board of Delta State University (Cleveland, MS). All women gave their written informed consent prior to study participation.

### Interventions

Both arms of the intervention were delivered in the participant’s home by trained Parent Educators. Parent Educators were African American, college educated women residing in the target communities. They were trained to deliver both the PAT and the PATE curriculum and to collect data from participants, including anthropometrics and dietary intake, by senior research staff members who were certified master trainers for the Nutrition Data System for Research (NDSR) software. Home visits occurred monthly and were approximately 60–90 min in length for the PAT arm and approximately 90–120 min for the PATE arm. Additional details regarding Parent Educator training, study methodology, and lesson plan outlines have been published elsewhere [20].

The PAT arm of the intervention was based on the PAT curriculum that included one-on-one home visits, optional monthly group meetings, developmental screenings, and a resource network for families. Using the PAT model, Parent Educators provided parents with research based information and activities during home visitation. Materials were responsive to parental information requests and were tailored to the age of the child (or gestational age of the fetus in the prenatal period).

The PATE arm of the intervention built upon the PAT curriculum. The curriculum enhancement was guided by the theoretical underpinnings of the social cognitive theory [24] and the transtheoretical model of behavior change [25]. Additionally, the PATE curriculum included foundational elements from the Diabetes Prevention Program and the Infant Feeding Activity and Nutrition Trial. Elements based upon the Diabetes Prevention Program included a culturally sensitive, individualized educational curriculum taught on a one-to-one basis [26]. Elements taken from the Infant Feeding Activity and Nutrition Trial included anticipatory guidance and parenting support principles [27]. Anticipatory guidance involves providing practical, developmentally appropriate, child health information to parents in anticipation of significant physical, emotional, and psychological milestones [28]. Parenting support emphasizes children’s psychological and behavioral goals, logical and natural consequences, mutual respect, and encouragement techniques [29].

For the PATE lessons, additional emphasis was placed on educating the women about ways in which they could develop positive eating, physical activity, and other health behaviors in their children, including modeling these behaviors themselves. Specific to the postnatal period, intervention components of the PATE arm included healthy weight management for mom, infant feeding cues, baby tummy time, introduction to solid foods for baby, healthy beverage selections for the family, sitting and screen time for mom and baby, modeling healthy behaviors for the child, creating a healthy home, healthy meal planning and food shopping, and toddler feeding. The entire perinatal lifestyle curriculum is available at: https://www.ars.usda.gov/ARSUserFiles/60000000/DeltaHumanNutritionResearch/DHS%20Lesson%20Plan%20Booklet.pdf.

Postnatal weight management for mothers, including tracking weight gain/loss, was discussed during the postnatal month (PM) 2–11 visits. At PM 2, women in the PATE arm were provided a US Department of Agriculture (USDA) MyPlate for Moms eating plan that was selected to promote 5% weight loss based on their PM 1 body weight while considering caloric needs if the woman was breastfeeding. For PATE participants, mean body weight at PM 1 was 9.3% above pre-pregnancy values. Women were already familiar with the USDA MyPlate eating plan approach given this tool was used to promote optimal gestational weight gain in the prenatal period [20, 21]. At the PM 3–11 visits, Parent Educators reviewed participants’ MyPlate diet and physical activity self-monitoring tracking logs, facilitated setting or revising eating and activity goals, and held discussions with participants regarding how to achieve their goals. Other curriculum features specific to maternal weight management included viewing the *How to Create a Great Plate* DVD (Learning Zone, 20 min) in PM 2, *Beverage Basics* DVD in PM 5 (Lemon-Aid Films, 8 min) and *Shop Healthy, Cook Healthy* DVD (Milner-Fenwick Inc., 16 min) in PM 7. At PM 2, 5, 7, and 9, dietary intake data that was collected during the previous month’s visit was reviewed with the participant. Parent Educators praised healthy food and beverage choices and discussed methods to amend food selections that were energy dense and nutrient poor. Women who were able to achieve the 5% weight loss goal in PM 3–11 were encouraged to maintain this weight loss through PM 12.

### Measures

#### Anthropometrics

Height at baseline was measured using a portable stadiometer (model 217, seca, Birmingham, UK). Maternal body weight was measured at baseline and every subsequent visit in the gestational and postnatal periods with an electronic scale (model SR241, SR Instruments, Tonawanda, NY). Pre-pregnancy body weight was self-reported. Body mass index was calculated as weight (kg) divided by height (m) squared.

#### Diet

Self-reported dietary intake data were collected from the participants at the PM 1, 4, 6, 8, and 12 visits via multiple pass 24-h dietary recall using NDSR software. NDSR is a Windows-based dietary analysis program that allows for the calculation of nutrients per ingredient, food, meal, and day in report and analysis formats [30]. Participants’ diet quality was calculated using the dietary data collected with NDSR and the Healthy Eating Index-2010 (HEI-2010) which measures adherence to the 2010 Dietary Guidelines for Americans [31].

#### Physical activity

Self-report postnatal physical activity data were collected from participants at the PM 1, 6, and 12 visits using a modified version of the Pregnancy and Physical Activity Questionnaire [32]. Modifications included small wording changes (e.g., driving or riding in a car vs. driving or riding in a car or bus) and timeframe adjustment (during this month vs. during this trimester) to make the instrument more relevant to this population of rural, Southern women and the Delta Healthy Sprouts Trial design. This 26-item instrument allows for the calculation of physical activity duration, intensity, specific type (i.e., sedentary, light-intensity, moderate-intensity, vigorous-intensity, household/care-giving, occupational, and sports/exercise), and total activity. Moderate and vigorous intensity physical activity responses were combined into a single category, moderate-to-vigorous physical activity (MVPA), because so few women reported time spent in vigorous activity.

Participants also provided information regarding demographic characteristics (e.g., age, marital status, household size, education, employment, household income, insurance, prenatal care), health history, and current health conditions at baseline (approximately 16 weeks gestation). Details regarding other measures and questionnaire data that were collected, but are not relevant to the present paper, have been published elsewhere [20]. All measures and questionnaires were collected or administered by trained research staff (Parent Educators) using laptop computers loaded with relevant software (i.e., Snap Surveys, NDSR) and in the participants’ homes.

### Statistical analyses

Because maternal postnatal weight control was the primary focus of this paper, analyses were conducted only for the postnatal cohort (participants who completed the gestational period and had at least one visit in the postnatal period; *n* = 54). Five participants who completed the gestational period but dropped out of the study prior to the PM 1 visit were excluded from the postnatal cohort. Additionally, one PAT participant who became pregnant again between the PM 1 and PM 2 visits was excluded from the weight control analyses. Similarly, visits occurring after conception for four PATE participants who became pregnant again between the PM 3 and PM 10 visits were excluded from the weight control analyses. Conception dates were determined by inputting participants’ reported due dates into an online pregnancy calculator [Pregnancy Calculator, http://www.calculator.net/pregnancy-calculator.html].

Statistical analyses were performed using SAS® software, version 9.4 (SAS Institute Inc., Cary, NC). Descriptive statistics, including means, standard deviations, frequencies, and percentages, were used to summarize participants’ demographic characteristics and anthropometric measures. Chi square tests of association or Fisher’s exact tests (categorical measures) and two sample t tests (continuous measures) were used to assess differences between PAT and PATE participants’ baseline, gestational, and some postnatal characteristics and measures. These tests also were used to assess differences between postnatal period study completers’ and non-completers’ baseline characteristics. Postnatal period study completers were defined as participants who had their PM 12 visit. Postnatal period study non-completers were defined as participants who had at least one visit in the postnatal period but did not complete the PM 12 visit.

Postnatal weight change was calculated using several methods. First, measured weight at each subsequent postnatal (PM 2 through PM 12) visit was subtracted from the measured weight for the PM 1 visit to obtain a postnatal difference value. Second, these difference values were divided by the PM 1 weight and then multiplied by 100 to obtain a postnatal weight change percentage. Third, self-reported pre-pregnancy weight was subtracted from measured weight at each postnatal (PM 1 through PM 12) visit to obtain a postnatal weight retention value. Fourth, these retention values were divided by the pre-pregnancy weight and then multiplied by 100 to obtain a postnatal weight retention percentage.

Linear mixed models, using maximum likelihood estimation, were used to test for significant treatment, time, and treatment by time (interaction) effects on postnatal weight outcomes. Maximum likelihood estimation is an approach for handling missing data in repeated measures. Treatment (PAT vs. PATE) was modeled as a fixed effect for all outcomes. Postnatal weight outcomes were modeled using a Gaussian (normal) distribution with an identity link function and time (PM1 through PM 12 visits) was modeled as a repeated measure using a variance covariance structure. Least squares means with 95% confidence limits were computed using these models. The first model included treatment, time, and treatment by time as fixed effects. The second model included pre-pregnancy BMI (continuous form) and treatment by BMI as additional covariates. The third model included only treatment as a fixed effect and was restricted to pre-pregnancy and PM 12 body weight data. This third model was run because our original hypothesis stated that the PATE participants would have less pregnancy weight retention at 12 months postnatal [Thomson CCT 2014). The significance level of the tests was set at 0.05.

## Results

Retention rates for the postnatal period for the PAT and PATE treatment arms were 83% (25/30) and 88% (21/24), respectively, and did not differ significantly between treatment arms (*p* = 0.668). The mean number of postnatal visits were 10.2 and 9.9 (*p* = 0.717), respectively, for PAT and PATE participants.

Table 1 presents comparisons between treatment arms for baseline socio-demographic characteristics of the postnatal cohort. Significant differences between PAT and PATE participant characteristics at baseline were not found with the exception of percentages receiving SNAP benefits. Significantly more PAT participants (87%) received SNAP benefits as compared to PATE participants (63%). The majority of both PAT and PATE participants were African American (approximately 96% in both groups) and reported single as their relationship status (87% vs. 92%). The mean age in the PAT group was 24.1 years and 23.0 years in the PATE group. Regarding completion status for the postnatal period, significant differences between completers and non-completers were not found for any of the baseline characteristics tested.

**Table 1:** Delta Healthy Sprouts participant baseline socio-demographic characteristics by treatment arm

	PAT (*N* = 30)		PATE (*N* = 24)		
Characteristic	*n*	%	*n*	%	*P*
Race					
African American	29	96.7	23	95.8	1.000
White	1	3.3	1	4.2	
Marital status					
Single^a^	26	86.7	22	91.7	0.682
Married	4	13.3	2	8.3	
Education level					
≤ High school graduate	12	40.0	12	50.0	0.462
≥ Some college/technical	18	60.0	12	50.0	
Employment status					
Full time/part-time	10	33.3	11	45.8	0.608
Unemployed (looking)	12	40.0	7	29.2	
Homemaker/student	8	26.7	6	25.0	
Smoker in household	7	23.3	9	37.5	0.257
Smoker^b^					
Current	1	3.3	1	4.2	0.620
Stopped before pregnancy	1	3.3	0	0.0	
Stopped after became pregnant	1	3.3	0	0.0	
Non	27	90.0	23	95.8	
Medicaid health insurance	30	100.0	24	100.0	0.703
Receiving SNAP	26	86.7	15	62.5	0.039
Receiving WIC	28	93.3	20	83.3	0.389
	Mean	SD	Mean	SD	*P*
Age (years)	24.1	4.76	23.0	4.96	0.380
Household size	3.6	1.61	4.2	1.52	0.221

*PAT* Parents as Teachers control treatment, *PATE* Parents as Teachers Enhanced experimental treatment, *SNAP* Supplemental Nutrition Assistance Program, *WIC* Special SNAP for Women, Infants and Children

^a^Included 1 participant who indicated she is divorced

^b^Comparison: non vs. all other responses

Table 2 presents comparisons between treatment arms for pre-pregnancy, pregnancy, and postnatal characteristics of the postnatal cohort. Significant differences between PAT and PATE participant characteristics were not found. Mean pre-pregnancy BMI in both treatment arms was in the overweight range (25.0–29.9 kg/m^2^). Mean gestational weight gain was approximately 15 kg in both groups with 53% of PAT participants and 71% of PATE participants gaining above the IOM recommendations for a singleton pregnancy. At the PM 1 visit, mean BMI in the PAT group was 30.4 ± 7.73 kg/m^2^ whereas in the PATE group, mean BMI was 31.6 ± 7.77 kg/m^2^. Overall, few women initiated and or sustained breastfeeding for more than 1 month. Mean HEI-2010 total score (not reported in table) for PAT participants was 40.2 and 36.4 at PM 6 and 12 while mean HEI-2010 total score for PATE participants was 40.2 and 37.6, respectively, and did not differ between the two groups (Thomson et al., under review). Further, women were well below population-level postpartum physical activity recommendations of 150 min/week [33]. Mean MVPA (not reported in table) for PAT participants was 50 min at both PM 6 and 12 while mean MVPA for PATE participants was 42 and 40 min at PM 6 and 12, respectively, and did not differ between treatment arms. (Thomson et al., accepted, American Journal of Health Promotion).

**Table 2:** Delta Healthy Sprouts participant pre-pregnancy, pregnancy and postnatal characteristics by treatment arm

	PAT (N = 30)		PATE (N = 24)		
Characteristic	*n*	%	*n*	%	*P*
Gestational diabetes	0	0.0	0	0.0	NA
Gestational hypertension	5	16.7	2	8.3	0.443
Gestational weight gain^a,b^					
Within IOM recommendations	9	30.0	2	8.3	0.087
Under IOM recommendations	5	16.7	5	20.8	
Above IOM recommendations	16	53.3	17	70.8	
Rate of gestational weight gain^b,c^					
Within IOM recommendations	5	16.7	3	12.5	0.720
Under IOM recommendations	6	20.0	4	16.7	
Above IOM recommendations	19	63.3	17	70.8	
Breastfeeding^d^					
> 1 month	2	6.7	2	8.3	1.000
< 1 month	7	23.3	10	41.7	
Never	21	70.0	12	50.0	
	Mean	SD	Mean	SD	*P*
Pre-pregnancy weight (kg)	76.4	22.10	80.0	24.78	0.566
Pre-pregnancy BMI	28.6	8.18	29.2	7.72	0.762
Gestational weight gain (kg)^a^	15.3	9.80	14.3	7.19	0.663
Postnatal weight (kg) at PM 1	81.6	22.48	86.4	24.74	0.460
Postnatal BMI at PM 1	30.4	7.73	31.6	7.77	0.577

*PAT* Parents as Teachers control treatment; PATE, Parents as Teachers Enhanced experimental treatment, *NA* Not applicable because all participants fall into single category, *IOM* Institute of Medicine, *BMI* Body mass index, *MVPA* Moderate to vigorous physical activity, *PM* Postnatal month

^a^Based on self-reported pre-pregnancy weight

^b^Comparison = within vs. under and above

^c^Based on measured weight between gestational months 4 and 9

^d^Comparison: > 1 month vs. < 1 month and never

Postnatal weight loss results (difference in kg or % body weight from PM 1 weight) are presented in Table 3. Mean weight losses for PAT participants were 0.8 and 1.1 kg at PM 6 and PM 12, respectively. Mean weight losses for PATE participants were 1.5 and 1.2 kg at PM 6 and PM 12, respectively. Significant effects were not found for treatment, time, or treatment by time. These results did not differ (i.e., no treatment effect) when only data for PM 12 were analyzed (*p* = 0.852).

**Table 3:** Delta Healthy Sprouts participant postnatal weight loss by treatment arm and visit (time)

	PAT (*n* = 29)^a^			PATE (n = 24)^b^			P		
Visit	LSM	95% CL		LSM	95% CL		Arm	Time	Int
Difference (kg) from PM 1 (negative = loss)									
PM 1	0.0	−1.12	1.12	0.0	−1.25	1.25	0.587	0.778	0.980
PM 2	−0.7	−1.83	0.48	−0.5	−1.88	0.78			
PM 3	−1.0	−2.26	0.18	−1.1	−2.49	0.39			
PM 4	−1.0	−2.24	0.26	−1.8	−3.28	−0.32			
PM 5	−0.9	−2.20	0.35	−0.6	−2.09	0.87			
PM 6	−0.8	−2.04	0.40	−1.5	−3.04	0.11			
PM 7	−0.8	−2.03	0.47	−0.8	−2.30	0.75			
PM 8	−1.2	−2.44	0.11	−1.0	−2.47	0.41			
PM 9	−0.9	−2.17	0.38	−0.2	−1.73	1.33			
PM 10	−1.3	−2.58	−0.03	−0.5	−2.05	1.01			
PM 11	−1.2	−2.45	0.04	0.1	−1.54	1.72			
PM 12	−1.1	−2.37	0.12	−1.2	−2.64	0.32			
% difference from PM 1 (negative = loss)									
PM 1	0.0	−1.39	1.39	0.0	−1.56	1.56	0.270	0.665	0.986
PM 2	−0.9	−2.33	0.56	−0.5	−2.22	1.12			
PM 3	−1.5	−3.03	0.03	−1.3	−3.08	0.52			
PM 4	−1.5	−3.10	0.02	−2.2	−4.02	−0.32			
PM 5	−1.5	−3.09	0.10	−0.7	−2.55	1.15			
PM 6	−1.4	−2.88	0.17	−2.0	−3.96	−0.02			
PM 7	−1.3	−2.82	0.29	−1.1	−3.05	0.77			
PM 8	−1.8	−3.38	−0.19	−1.4	−3.22	0.38			
PM 9	−1.3	−2.85	0.34	−0.4	−2.31	1.51			
PM 10	−1.8	−3.36	−0.18	−0.4	−2.36	1.46			
PM 11	−1.6	−3.20	−0.08	0.0	−2.01	2.07			
PM 12	−1.5	−3.04	0.08	−1.2	−3.03	0.68			

*PAT* Parents as Teachers control treatment, *PATE* Parents as Teachers Enhanced experimental treatment, *LSM* Least squares mean, *CL* Confidence limit, *Int* Interaction, *PM* Postnatal month

^a^Excluded post conception visits for 1 PAT participant who became pregnant again in postnatal period

^b^Excluded post conception visits for 4 PATE participants who became pregnant again in the postnatal period

Postnatal weight retention (difference in kg or % body weight from self-reported pre-pregnancy weight) results are presented in Table 4. Mean weight retention for PAT participants was 5.2, 4.0, and 3.6 kg at PM 1, PM 6, and PM 12, respectively. Mean weight retention for PATE participants was 6.3, 4.5, and 4.0 kg at PM 1, PM 6, and PM 12, respectively. Significant effects were not found for treatment, time, or treatment by time. Again, these results did not differ (i.e., no treatment effect) when only data for PM 12 were analyzed (*p* = 0.790).

**Table 4:** Delta Healthy Sprouts participant postnatal weight retention by treatment arm and visit (time)

	PAT (n = 29)^a^			PATE (n = 24)^b^			P		
Visit	LSM	95% CL		LSM	95% CL		Arm	Time	Int
Difference (kg) from pre-pregnancy weight (positive = retain)									
PM 1	5.2	2.53	7.89	6.3	3.32	9.31	0.390	0.982	1.000
PM 2	4.7	1.94	7.48	5.0	1.84	8.23			
PM 3	3.9	0.94	6.81	5.2	1.72	8.63			
PM 4	3.9	0.88	6.86	3.5	−0.05	7.06			
PM 5	3.9	0.81	6.92	4.7	1.11	8.22			
PM 6	4.0	1.09	6.96	4.5	0.74	8.31			
PM 7	4.0	0.99	6.98	3.9	0.23	7.56			
PM 8	3.5	0.48	6.59	3.7	0.26	7.17			
PM 9	3.6	0.59	6.70	4.4	0.71	8.04			
PM 10	3.2	0.17	6.29	4.3	0.63	7.96			
PM 11	3.6	0.56	6.55	4.7	0.74	8.58			
PM 12	3.6	0.65	6.63	4.0	0.49	7.60			
% difference from pre-pregnancy weight (positive = retain)									
PM 1	7.7	4.47	10.87	9.3	5.71	12.86	0.683	0.649	0.999
PM 2	6.9	3.59	10.22	7.1	3.26	10.91			
PM 3	5.7	2.22	9.23	7.1	2.96	11.22			
PM 4	5.5	1.95	9.10	4.0	−0.23	8.27			
PM 5	5.7	2.09	9.39	5.2	0.97	9.47			
PM 6	5.7	2.17	9.18	4.9	0.36	9.41			
PM 7	5.8	2.18	9.34	4.0	−0.43	8.34			
PM 8	5.0	1.36	8.66	4.0	−0.13	8.13			
PM 9	5.5	1.84	9.15	5.0	0.57	9.33			
PM 10	5.0	1.34	8.65	4.7	0.37	9.13			
PM 11	5.3	1.76	8.91	5.3	0.63	9.99			
PM 12	5.5	1.93	9.08	4.8	0.54	9.04			

*PAT* Parents as Teachers control treatment, *PATE* Parents as Teachers Enhanced experimental treatment, *LSM* Least squares mean, *CL* Confidence limit, *Int* Interaction, *PM* Postnatal month

^a^Excluded post conception visits for 1 PAT participant who became pregnant again in postnatal period

^b^Excluded post conception visits for 4 PATE participants who became pregnant again in postnatal period

Pertaining to the results for which pre-pregnancy BMI and its interaction with treatment arm were included as covariates, only pre-pregnancy BMI was significant for the postnatal weight loss outcome models, although its effect was small [slope = 0.1, standard error (SE) = 0.03, *p* = 0.002 for kg difference; slope = 0.1, SE = 0.04, *p* = 0.001 for percent difference). For the postnatal weight retention (kg) model, treatment and its interaction with pre-pregnancy BMI were significant (*p* < 0.001 for both). Specifically, the slope for pre-pregnancy BMI was 0.1 (SE = 0.07) for the PATE treatment arm, while the slope was −0.3 (SE = 0.09) for the PAT treatment arm. That is, for every 1-unit increase in pre-pregnancy BMI, retained weight increased by 0.1 kg for PATE participants, while retained weight decreased by 0.3 kg for PAT participants.

Somewhat similarly, for the postnatal weight retention (percent) model, treatment, pre-pregnancy BMI, and their interaction term were significant (p = 0.001, < 0.001, and <0.001, respectively). Specifically, the slope for pre-pregnancy BMI was −0.1 (SE = 0.08) for the PATE treatment arm, while the slope was −0.5 (SE = 0.10) for the PAT treatment arm. That is, for every 1-unit increase in pre-pregnancy BMI, retained weight decreased by 0.1% for PATE participants, while retained weight decreased by 0.5% for PAT participants.

## Discussion

This paper reports on the treatment effect differences for postnatal weight change and weight retention through 12 months postpartum for women enrolled in the Delta Healthy Sprouts Trial. This trial is one of only a few trials to conduct a maternal weight management intervention targeting both the gestational and postpartum periods in the context of a single intervention [15–19]. Findings from this analysis indicate that participants in the PATE experimental arm did not lose more weight in the postpartum (between PM 1 and PM 12) or retain less weight gained in pregnancy compared to the women in the PAT control arm.

The findings of our study are similar to two other maternal weight management interventions that spanned the perinatal period. In the New Life(style) study conducted by Althuizen and colleagues [17, 34], women received in-person counseling from a midwife to optimize weight gain in the gestational period and one telephone counseling session at 8 weeks postpartum to promote weight loss [34]. They reported no significant effect of the intervention on maternal weight at 1 year postpartum compared to a control group. In the Trial for Reducing Weight Retention in New Moms [19], women randomized to the enhanced care arm received weight loss/behavior change concepts delivered through a single in-person nutrition counseling session and monthly newsletters. There was no difference in weight loss or weight retention between the intervention and standard care group which received information about nutrition guidelines for breastfeeding at 6 months postpartum. However, it is important to highlight that despite similarities in findings, our intervention was longer in duration and more intensive.

In contrast to our study, a perinatal intervention conducted with Taiwanese women [16] reported that women receiving a combined gestational and postpartum weight management intervention retained less weight at 6 months postpartum compared to women receiving only the postpartum intervention or control treatment. In a study conducted by Clasesson and colleagues [15], women receiving weekly weight gain optimization support during pregnancy and every 6 months through 2 years postpartum to promote weight change had significantly greater weight loss compared to a standard care control group. Liu et al. [18] conducted a small pilot study with intervention components comparable to Delta Healthy Sprouts (e.g., used USDA MyPlate for Moms to promote weight management) in a similar population of pregnant Southern, African American women. The gestational intervention involved one face-to face individual meeting and eight group sessions, while the postpartum intervention included one home visit and three telephone-based sessions. At 12 weeks postpartum, 50% of their postnatal cohort was at their pre-pregnancy body weight or lower. However, the authors did not compare these postpartum weight findings against a control group and the sample size of the postnatal cohort was only 14 women.

Several other studies have focused their intervention exclusively on the postpartum period. In a recent systematic review of 11 lifestyle interventions to limit postpartum weight retention [35], seven of the 11 studies were successful at promoting postpartum weight loss. Of the seven successful trials [16, 36–41], six [16, 36–40] incorporated both dietary and physical activity components. Although the dietary components used in these trials were similar to the Delta Healthy Sprouts Trial, there was clearly a greater emphasis placed on increasing postpartum physical activity with some trials including supervised physical activity sessions [36, 38] and the provision of heart rate monitors [36, 41]. The majority of the successful trials also tended to engage with participants on a more frequent basis (i.e., more than monthly). Thus, a greater emphasis on physical activity and more frequent participant contact may have increased the efficacy of our postpartum PATE intervention.

Interestingly, based on our linear mixed models analyses, the PATE treatment appeared more effective in terms of postnatal weight loss for participants with lower pre-pregnancy BMI, while the PAT treatment appeared more effective for participants with higher pre-pregnancy BMI. We also observed that both PATE and PAT treatments appeared more effective in terms of producing a lower percentage of weight retention for participants with higher pre-pregnancy BMI, although the effect was more pronounced in the PAT participants. These findings are difficult to interpret but suggest that the women with pre-pregnancy obesity were more successful with postpartum weight management when it was self-directed vs. through a lifestyle intervention explicitly focusing on postnatal weight management.

Our overall lack of intervention effect could be due to the complexity of our intervention. To promote postpartum weight change in the PATE arm, we recommended following a personalized USDA MyPlate for Moms eating plan designed to produce about a ½ -1 lb of weight loss per week. This approach combined both energy restriction and improving overall diet quality. Leermarkers et al. [39] found a significant effect on postpartum weight loss in a low-intensity intervention that focused exclusively on reducing overall calories. Thus, it is possible that simultaneously targeting multiple dietary behaviors was overwhelming to our participants. Bennett and colleagues [42] have suggested that health literacy may complicate weight management for medically vulnerable populations. He suggests that promoting weight management through easy to understand dietary behavior goals (i.e., reducing sugary beverages) may be more effective for weight management for persons with low health literacy. Although we did not examine health literacy in the context of the Delta Healthy Sprouts Trial, there was relatively low educational attainment in 50% of the PATE and 40% of the PAT women. Thus, future lifestyle interventions targeting this population of pregnant women should consider the health literacy of participants [42].

Phelan and colleagues suggest that the success of an intervention focused on reducing postpartum weight retention is largely dependent on the ability to optimize weight gain in the gestational period [43]. Almost three-fourths of the women in the PATE treatment arm exceeded the IOM recommendations for gestational weight gain. Furthermore, approximately 50% of the women initially enrolled in our trial exceeded the IOM weight gain recommendations in their fourth month of pregnancy [44]. Thus, interventions targeting women earlier in pregnancy (i.e., 6–10 weeks gestation) may allow for more favorable postpartum weight outcomes given that gestational weight gain is a significant predictor of postpartum weight retention [45].

There are strengths and weaknesses in this study. The longitudinal design is one of its greatest strengths given women were followed through 12 months postpartum. Further, the population studied is a strength given that Southern, African American women are at increased risk for obesity and chronic diseases [9]. Our study also was personalized [16], home-based [46], built upon a known national MIECHV program [47], and theory-driven [18], all of which have been cited as salient features for lifestyle interventions targeting pregnant women. A significant limitation of our study was the high level of attrition observed in both PAT and PATE treatment arms (58 and 54%, respectively) from baseline to study end which is higher than dropout reported in similar trials [15–19, 46]. Our small sample size resulting from high attrition may have been a limiting factor in detecting statistically significant differences between the two treatment arms. Additionally, data collection was not blinded and therefore is a potential source of bias. However, having a second set of blinded research staff whose sole purpose was to collect data was not practically, logistically, or financially feasible. Moreover, it is unlikely that bias occurred on the part of the Parent Educators or the participant (e.g., provision of socially desirable responses) given the lack of effect observed in this study. Another limitation was the use of self-report measures, including pre-pregnancy body weight, which could have biased our estimation of gestational weight gain and postpartum weight retention.

## Conclusions

Our lifestyle-enhanced MIECHV curriculum was not associated with more favorable postpartum weight outcomes when compared to a standard MIECHV curriculum in a cohort of postpartum LMD African American women. Weight management in the postpartum remains a significant public health concern given that retaining an excessive amount of the weight gained in pregnancy can compromise a woman’s future reproductive health [6, 7] and increase her risk for chronic health conditions [4, 5]. Future studies targeting lifestyle behaviors of pregnant and postpartum women in the health disparate LMD region should consider placing a greater emphasis on increasing physical activity, inclusion of simplified dietary messaging to accommodate women with lower levels of health literacy, and increased frequency of contact with participants, particularly in the gestational period.
